# Molecular Differentiated Initiator Reactivity in the Synthesis of Poly(caprolactone)-Based Hydrophobic Homopolymer and Amphiphilic Core Corona Star Polymers

**DOI:** 10.3390/molecules201119681

**Published:** 2015-11-09

**Authors:** Eileen Deng, Nam T. Nguyen, Frédéric Hild, Ian E. Hamilton, Georgios Dimitrakis, Samuel W. Kingman, Phei-Li Lau, Derek J. Irvine

**Affiliations:** 1 Department of Chemical and Environmental Engineering, Faculty of Engineering, University of Nottingham, Nottingham NG7 2RD, UK; dengeileen@hotmail.com (E.D.); nguyen.nam@hotmail.co.uk (N.T.N.); frederichild@gmail.com (F.H.); I.Hamilton@bham.ac.uk (I.H.); enzgd1@exmail.nottingham.ac.uk (G.D.); Sam.Kingman@nottingham.ac.uk (S.W.K.); 2 School of Chemistry, University of Nottingham, Nottingham NG7 2RD, UK; 3 Department of Chemical and Environmental Engineering, University of Nottingham Malaysia Campus, Jalan Broga, Semenyih 43500, Selangor Darul Ehsan, Malaysia; Phei-Li.Lau@nottingham.edu.my

**Keywords:** ring opening polymerisation, microwaves, star polymer, core corona star, dielectric properties

## Abstract

Macromolecules that possess three-dimensional, branched molecular structures are of great interest because they exhibit significantly differentiated application performance compared to conventional linear (straight chain) polymers. This paper reports the synthesis of 3- and 4-arm star branched polymers via ring opening polymerisation (ROP) utilising multi-functional hydroxyl initiators and Sn(Oct)_2_ as precatalyst. The structures produced include mono-functional hydrophobic and multi-functional amphiphilic core corona stars. The characteristics of the synthetic process were shown to be principally dependent upon the physical/dielectric properties of the initiators used. ROP’s using initiators that were more available to become directly involved with the Sn(Oct)_2_ in the “*in-situ*” formation of the true catalytic species were observed to require shorter reaction times. Use of microwave heating (MWH) in homopolymer star synthesis reduced reaction times compared to conventional heating (CH) equivalents, this was attributed to an increased rate of “*in-situ*” catalyst formation. However, in amphiphilic core corona star formation, the MWH polymerisations exhibited slower propagation rates than CH equivalents. This was attributed to macro-structuring within the reaction medium, which reduced the potential for reaction. It was concluded that CH experiments were less affected by this macro-structuring because it was disrupted by the thermal currents/gradients caused by the conductive/convective heating mechanisms. These gradients are much reduced/absent with MWH because it selectively heats specific species simultaneously throughout the entire volume of the reaction medium. These partitioning problems were overcome by introducing additional quantities of the species that had been determined to selectively heat.

## 1. Introduction

Research focused on broadening the range of molecular structures exhibited by biodegradable/bioresorbable aliphatic polyesters has atttracted an increased level of interest in recent decades. This has often been focused upon improving the viability of such materials as alternatives to petrochemical-based polymers in a large variety of end-uses. In the particular cases of poly(lactic acid) (PLA) and poly(ε-caprolactone) (PCL), this interest has typically been inspired by their utilisation in biomedical and pharmaceutical applications [1,2,3,4,5,6,7].

**Figure 1:**
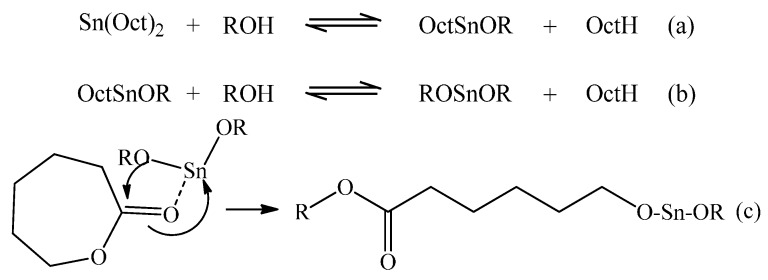
Mechanism of initiation of ROP using Sn(Oct)_2_ as the precatalyst and a mono-functional alcohol initiator.

In this area, ring-opening polymerisation (ROP) of cyclic esters (e.g., dilactide, ε-caprolactone, *etc.*) using discrete organometallic based initiators/catalyst systems has become established as one of the preferred methods of generating well-defined and potentially stereoregular polyester molecular structures with narrow polydispersities (PDI) [8,9,10,11,12,13,14,15,16,17]. Generally, this molecular control is achieved through precise control of the propagation and termination processes within the synthetic mechanisms involved in the polymerisation [8,9,10,11,12,13,14,15,16,17]. Additionally, the synthesis of biodegradable or bioresorbable star polyesters has drawn particular interest because of the differentiated rheological and mechanical properties that these structures can exhibit, when compared to linear polymers [18,19,20,21,22,23,24,25,26,27,28,29,30,31,32]. For example, the melt viscosity of star polymers have been reported to be lower than their linear analogues of a similar molecular weight (Mwt), so allowing moulding to be conducted at lower temperatures [6,33]. Therefore, it should be preferable to employ star structures when melt processing/thermally moulding, polymers such as PCL which exhibit relatively low thermal stability and/or molecular integrity. Typically, the synthesis of star polymers can be achieved by adopting one of three different approaches [18]. These are: (a) Core-first—polymerisation to build the arms is conducted away from a multifunctional initiator, which then forms the core of the structure; (b) Arm-first—coupling of pre-synthesized linear polymers with a multifunctional terminator or pre-existing multi-functional reactive species which becomes the core and (c) Direct Oligomerisation—controlled polymerisation of preformed macromonomers which contain a polymerisable functional group to a low/intermediate degree of polymerisation (Dp), the backbone of which forms the core of the structure (DP) [18,27]. For biodegradable polymers, such as PLA and PCL, the “core first” approach has been the most extensively employed method detailed in the literature, with the use of many different structures and functionalities of core molecules reported to successfully result in star structures [18]. For example, pentaerythritol (PTOL), glycerol (G), and 2-ethyl-2-hydroxymethyl-1,3-propanediol (or trimethylolpropane, TMP) have been successfully used as core initiators for the synthesis of stars via ROP using Sn(Oct)_2_ as a precatalyst [20,21,26,27,28]. Figure 1 depicts this generally accepted mechanism for ROP of CL with Sn(Oct)_2_ and benzyl alcohol (BzOH).

However, it has been reported that with multi-hydroxyl group containing initiators, *i.e*., the type which generate of star structures, that significant lengthening of the reaction time and in certain cases lower quality product (*i.e.*, broader PDIs and non-optimal Mwts) has been observed, when compared to the synthesis of equivalent length linear polymers under the same conditions [34]. This has been attributed to a number of potential root causes; (a) multi-ols have the potential to create more stable, multi-dentate complexes with the tin centre; (b) more Sn species need to coordinated to the initiator if all the chains are to grow simultaneously and (c) if arm growth is not simultaneous/uniformly from the outset, steric effects may slow the reaction at some functional groups in a particular initiator moiety [34]. Research investigating the use of other controlled polymerisation systems to create 3-dimensional structures has also shown differential reactivity of hydroxyl functionalities. For example work to prepare materials for surface initiated atom radical polymerisation have been shown to demonstrate variable hydroxyl group reactivity levels based on both structural and morphological issues, *i.e.*, low surface areas and low levels of site accessibility. This was mitigated by expanding the structure to overcome the steric hindrance around the reactive sites [35].

Consequently, the initial aim of this work was to focus upon the study of the “core-first” method of star polymer production using ROP with Sn(Oct)_2_ as the precatalyst. The aim was to investigate the influence that the use of multi-functional initiators with differing molecular/physical properties had upon the polymerisation kinetics and final products when different molecular weight homo- and amphiphilic core corona star polymer structures were synthesised. Also, because in a prior paper the authors had demonstrated differing initiation kinetics with single hydroxyl initiators when microwave heating (MWH) was adopted [34], equivalent CH and MWH experiments were conducted to highlight if different initiation behaviour was also observed with these more complex initiating species.

## 2. Results and Discussion

**Scheme 1:**
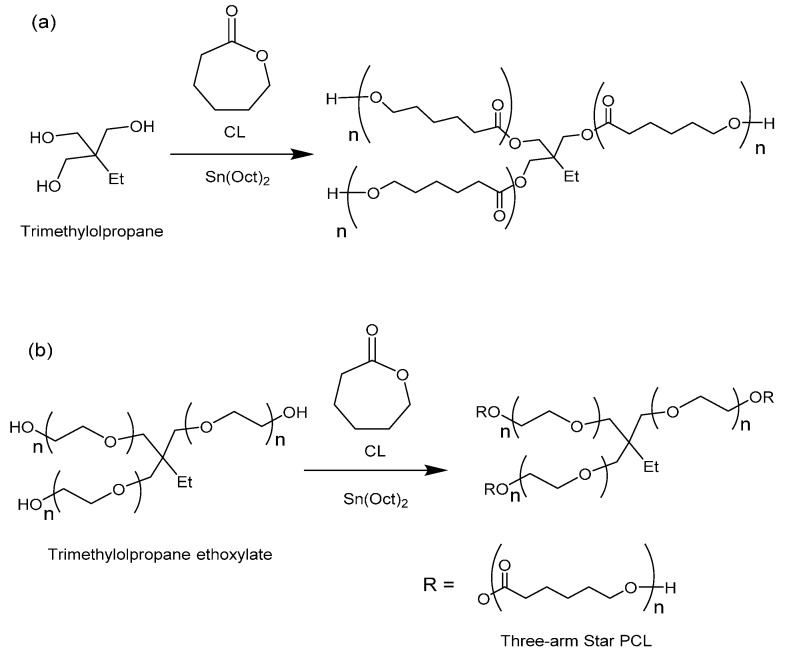
Synthesis of (**a**) hydrophobic homopolymer 3-arm stars using TMP as initiator and (**b**) amphiphilic 3-arm core corona stars using TMPE as initiator.

The synthesis of 3-arm star PCL hydrophobic homopolymers was initially conducted using trimethylolpropane (TMP) as a tri-functional initiator with both MWH and CH at 150 °C (see Scheme 1a) and compared BzOH initiated linear polymerisations.

These polymerisations were conducted in the bulk (*i.e.*, no solvent), both to maximise the polymerisation rate and because it has been reported that the use of significant amounts (*i.e.*, >40%) of organic solvent resulted in the loss of any MWH differences obtained [36]. Two relative CL/TMP molar ratios were used (*i.e.*, 21:1 and 90:1) to obtain polymers with predicted DP’s of 21 and 90 respectively (*i.e.*, theoretical arms of DP 7 and 30) to highlight the differences that target Mwt made to initiation with multi-ols. The relative molar ratio CL/Sn(Oct)_2_ was kept constant at 1:2.80 × 10^−4^. Table 1 summarises the reaction characteristics and material analysis results obtained.

**Table 1:** Results of CH & MWH ROP to Synthesise Hydrophobic Homopolymer Initiated by TMP, PTOL & Glycerol (G) with CL/Sn(Oct)_2_ ratio of 1:2.80 × 10^−4^ at 150 °C.

Entry	Initiator	Target DP	Heat Type	Time (min)	M_*n*(*theo*)_ (g·mol^−1^)	M_*n*(*GPC*)_^a^ (g·mol^−1^)	PDI ^a^
1	TMP	90	CH	150	10,300	6500	1.19
2	TMP	90	MWH	90	10,300	6500	1.24
3	TMP	21	CH	250	2400	1600	1.38
4	TMP	21	MWH	190	2400	1600	1.43
5	PTOL	88	CH	210	10,000	5100	1.21
6	PTOL	88	MWH	170	10,000	5100	1.23
7	PTOL	20	CH	390	2300	1100	1.58
8	PTOL	20	MWH	250	2300	1100	1.63
9	G	90	CH	45	10,300	13,500	1.37
10	G	90	MWH	35	10,300	15,300	1.23
11	G	21	CH	90	2400	6100	1.15
12	G	21	MWH	60	2400	4000	1.23

^a^ Measured by GPC in THF (40 °C) using PS standards and corrected by applying the correction factor for PCL (0.56) [37].

**Figure 2:**
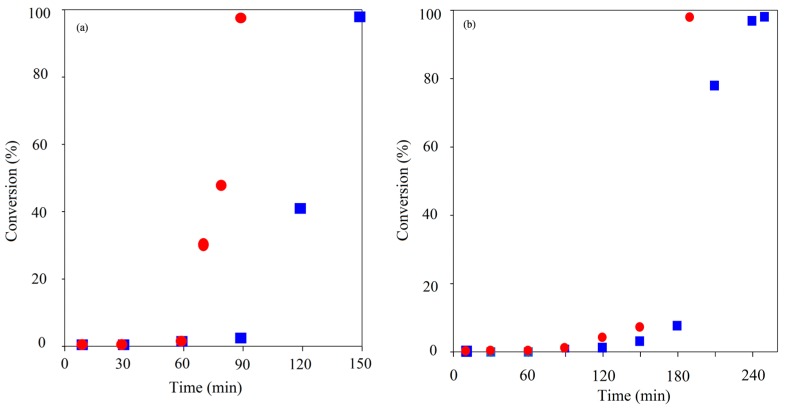
Comparison of TMP initiated ROP kinetics using CH (■) & MWH (●) at 150 °C and target DP 90 (**left**) and 21 (**right**).

The data in Table 1 showed that, in all cases, polymers with similar chain length and PDI’s were synthesised using both heating methods. However, the observed chain lengths were noted to be shorter than theoretically expected. This was attributed to two factors: (1) chain length determination for star polymers is inaccurate because their hydrodynamic volumes are different from that of the linear GPC standards [38] and (2) ROP reactions using this control system can undergo transesterifications at high conversions [39]. However, when this MWH data were compared to the reported data for linear polymers synthesised using BzOH as initiator [34], it was observed that the star polymers required significantly longer reaction times (for DP = 90: linear = 20, 3-arm = 90, 4-arm = 170 min) but the low PDI’s of the resultant polymers coupled, with the observation that all the GPC spectra contained monomodal distributions (Figure S1), indicated good polymerisation control had been achieved. Meanwhile, the application of MWH had decreased the reaction time required to synthesise both DP 90 and DP 21 materials to >95% conversion (compare Table 1, entries 1 & 2 and 3 & 4). Thus, MWH and CH kinetic experiments were conducted (see Figure 2) to define the root cause of the longer cycle times.

As with linear ROP reactions conducted using this system the kinetic pathway was shown to exhibit two stages: (a) an induction period, which represents the combination formation of the true catalyst by reaction of the Sn(Oct)_2_ with the initiator and the initiation with the first monomer unit and (b) the propagation stage. In all cases the propagation stage of the polymerisation was observed to be rapid, indicated by the significant gradient of the linear relationships exhibited once conversion has started. The key element that has been lengthened is the induction period. Furthermore, the induction period for the DP 21 IP was noted to be longer than that for DP 90 which was attributed to the need for greater levels of initiation required prior to propagation commencing. The principle influence of adopting. MWH with TMP under these conditions was to reduce the length of induction, which was typically reduced by 30 min (*i.e.*, 90 to 60 for DP 21 & 180 to 150 for DP 90).

Kinetic experiments to synthesise 4-arm star hydrophobic, homo-polymer PCL structures of DP 20 and 88 (arms of DP 5 and 22 respectively) were also conducted using a tetrafunctional initiator, pentaerythritol (PTOL) and the same reaction conditions (see Scheme S1 and Table 1, entries 5–8). The comparative characteristics of these reactions/products were observed to be the same as the 3-arm case: *i.e.*, the induction period was determined to represent the major element of the overall reaction time, smaller stars were observed to need longer reaction times, the application of MWH led to a decrease in both induction time/overall reaction times required for both DPs and the heating method was shown not to affect the structure of the product materials obtained, single distribution GPC spectra with equivalent Mwts and PDI’s were obtained for all isolated product polymers (see Figure S2). Subsequent kinetic experiments confirmed that the IP reduction was the key effect of the MWH upon the system. However, in this case, whilst the induction for DP 88 IP was reduced by 30 min (150 to 120), similar to that observed with TMP (see Figure S3). The DP 20 reduction was typically found to be reduced by 145 min (315 to 170 min). Thus, it was concluded from these experiments that the MWH reduction in IP was typically in the region of 20%–50%. However, MWH was noted to deliver smaller IP reductions with multi-ols (for DP 90: linear = 95%, 3-arm = 33%, 4-arm = 20%). Therefore, whilst these results demonstrated that MWH could overcome the issues of using multifunctional initiators with Sn(Oct)_2_, such as retardation due to chelation stabilisation. Therefore, both the fact that initiation is the dominant factor in determining reaction time and the relatively modest MWH differentials observed with multi-ols compared to mono-functional initiators required further investigation to understand the true influence of these initiators on the polymerisation.

Therefore, the influence that initiator physical property characteristics had upon defining the induction period was investigated. Thus, the physical form, solubility characteristics and loss tangent (tanδ) values (see dielectric property definitions in the experimental section) of the multi-ols used in this study and BzOH have been compared in Table 2.

**Table 2:** Physical Characteristics of the Initiators Used in this Study.

Initiator	Mol Weight (g·mol^−1^)	Melting Point (°C)	Physical Form ^c^	Solvent/Monomer Compatibility ^c^	Tanδ at 150 °C
BzOH	108.14	−15	Liquid	Miscible	0.40
TMP	134.17	58	Solid (Flake)	Limited	0.20
PTOL	136.15	260	White Solid	Very Limited	0.01
TMPE	~450 ^a^	NA ^b^	Liquid	Very Limited	-
PTOLE	~270 ^a^	NA ^b^	Liquid	Very Limited	-
G	92.09	18	Liquid	Miscible	0.25
GE	~1000 ^a^	NA ^b^	Liquid	Miscible	0.10

^a^ As quoted by supplier; ^b^ Data not available from supplier; ^c^ Data refers to a room temperature evaluation.

The physical form/miscibility data were determined in order to define if the length of the induction period was related to the actual availability of the initiator within the system, whilst the loss tanδ values were measured to predict the relative ability of specific materials to contribute to the heating of a mixture by being selective heated by microwave energy.

**Figure 3:**
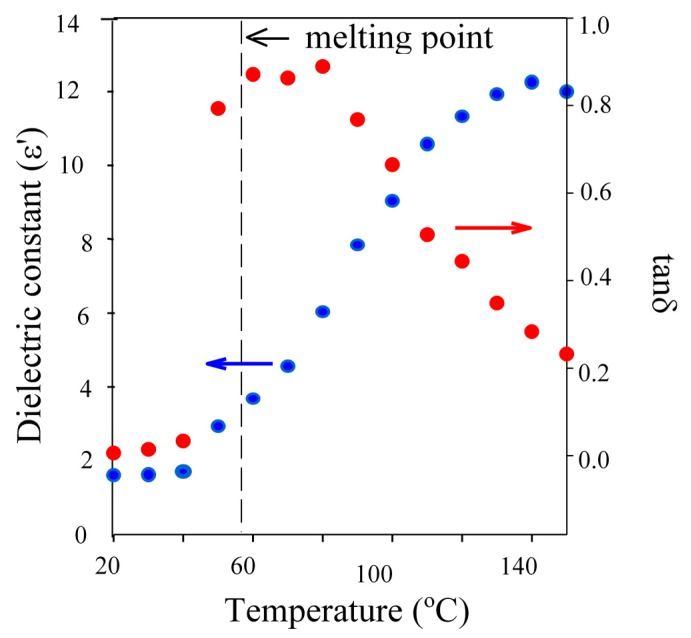
Plot of dielectric constant (●) and tanδ (●) against temperature for TMP demonstrating the influence of the melting transition on dielectric properties.

Interestingly, the observed trends in the length of both IPs and CTs when using BzOH (1 & 20 min), TMP (60 & 90 min) and PTOL (120 & 170 min), were consistent with the trends in their melting points, monomer compatibilities and tanδ properties. Both TMP and PTOL are solids at room temperature and not completely miscible with CL. Therefore, if the initiator and Sn precatalyst are not in the same phase, their interaction to form the “true” catalytic species will be hindered [34]. Whilst, comparison of dielectric data (Table 2 and Figure 3) showed that, at reaction temperature, the tanδ of TMP and PTOL were approximately 0.2 and 0.01 respectively, compared to 0.4 for BzOH.

BzOH was well above its melting point at in the range from room temperature to the set reaction temperature (150 °C) and very miscible with the organic monomer of choice, thus is able to interact with both the incident energy and the Sn precatalyst. However, whilst the TMP is also well above its melting point at 150 °C, it was observed to be less miscible with the monomer over the full temperature range which has related to its melting point and the presence of additional hydroxyl functionality. This results in it being less available to take part in the initiation or to be selectively heat heated by microwave energy because it is not fully “liquefied”. This can be seen in the rapid increase in it dielectric properties upon exceeding its melting point. Finally, at 150 °C, PTOL was observed to be below its melting point, exhibit limited miscibility with the monomer and thus yielded a tanδ value an order of magnitude lower than both BzOH and TMP. This identified PTOL as being much less available to interact with the tin precatalyst and also more microwave transparent at the applied reaction temperature and thus much less likely to undergo selective heating and so is far less able to become fully solvated when MWH was adopted thus it will be less able to readily overcome the issues related to multi-ol initiation, such as chelation. However, as the MWH and CH reaction profiles showed that, regardless of the DP, both initiation and propagation periods were shorter when using MWH for TMP and PTOL (see Figure 2 and Figure S3), it was concluded that the MWH was still achieving some level of selective heating within the reaction medium in all cases.

Thus the physical properties of the initiator was concluded to be a major influence over the level of the induction period with both heating methods as the reaction progresses over the temperature range from room to the set reaction temperature. Thus, the influence of the MWH will be to raise the reaction temperature more quickly than the conventional system so that the initiator becomes more compatible more quickly. This rapid heating of the system has been attributed to the presence of the tin species in the system, as proposed by Irvine and co-workers, in reactions to produce linear polymers at 150 °C [34]. This work demonstrated that adding catalytic amounts of Sn(Oct)_2_ to a sample of monomer resulted in the system both reaching the target temperature more quickly and achieving a final bulk temperature that was 20 °C higher than the intended target when MWH used [34]. Therefore, it was concluded that selective heating of the tin species makes the most significant contribution to overcoming convection and dilution issues encountered with star polymer reductions even when the initiator material properties do not favour MWH.

**Figure 4:**
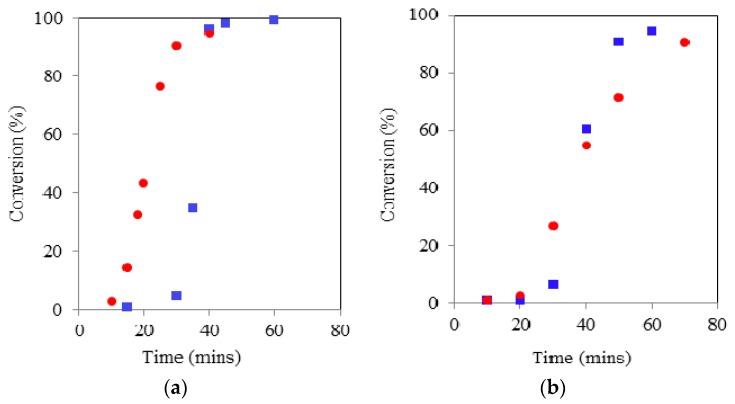
Comparison of G (**a**) and Glycerol Ethoxylate (GE) (**b**) initiated ROP the kinetics using CH (■) and MWH (●) (150 °C, DP90).

To investigate this physical form hypothesis further, the use of liquid multi-ol initiators was investigated. This should enhance both the initiator’s miscibility of the initiator in the monomer and susceptibility to microwave selective heating. Initially a ROP synthesis to form 3-arm star PCL hydrophobic homo-polymer was performed using G as the initiator. G exhibits both liquid physical form at room temperature and contained similar central hydrocarbyl section of the core structure to TMP, so its use would further probe if the elongated induction periods with TMP/PTOL were more influenced by the physical/dielectric properties of the initiator, see Table 1, entries 9–12 and Figure 4a.

All the ROP’s using G as the liquid physical form initiator gave both shorter induction and overall reaction times compared to those exhibiting solid form, e.g., for the CH reaction these times were 30 and 40 min respectively. This supported the conclusion that the physical form of the initiator has a large influence over the induction period observed and thus total cycle time required to complete the ROP. Furthermore, the cycle times of the G based MWH ROPs were significantly shorter than for the CH equivalents (*i.e.*, >10 min), supporting the conclusion that the MWH reductions are related to the selective heating of the tin species and the availability of the initiator because the tanδ of G is very similar to TMP.

The investigation of the influence of initiator physical form was then extended to include the synthesis of star-shaped polymers using liquid initiators which were the ethoxylated equivalents of TMP, PTOL, and G; *i.e.*, trimethylolpropane ethoxylate (TMPE), pentaerythritol ethoxylate (PTOLE) and glycrol ethoxylate (GE). These contain partially polymerised arms consisting of a low number (*i.e.*, 3–4 units) poly(ethylene glycol) (PEG) repeat units on each arm of a TMP, PTOL and G core. The addition of the ethoxylate chain ensures that these initiators now exhibit liquid physical form. Furthermore, their use will result in the synthesis of amphiphilic core corona star polymers with a hydrophilic core (PEG) and a hydrophobic outer corona (PCL). The molecular structures of these species are represented in Scheme 1b (TMPE) and Scheme S2 ESI (PTOLE). It was proposed that the adoption of these liquid initiators would both reduce the influence of the initiator’s physical form/miscibility upon the initiation period and reduce the level of steric crowding by increasing both flexibility in the initiator’s structures and the intermolecular distance between the hydroxyl functional groups. The results from these reactions are summarised in Table 3.

**Table 3:** Results for TMPE, PTOLE & GE initiated ROP to Synthesis Amphiphilic Core Corona Stars using CH & MWH with CL/Sn(Oct)_2_ = 1:2.80 × 10^−4^ at 150 °C.

Entry	Initiator	Target DP	Heat Type	Time ^b^ (min)	M_*n*(*theo*)_ (g·mol^−1^)	M_*n*(*GPC*)_^a^ (g·mol^−1^)	PDI ^a^
1	TMPE	90	CH	50	10,700	13,500	1.24
2	TMPE	90	MWH	50	10,700	13,600	1.21
3	TMPE	21	CH	60	2800	2300	1.33
4	TMPE	21	MWH	60	2800	2400	1.37
5	PTOLE	88	CH	60	10,500	14,400	1.27
6	PTOLE	88	MWH	60	10,500	14,800	1.30
7	GE	90	CH	50	11,300	12,300	1.31
8	GE	90	MWH	70	11,300	12,200	1.27
9	GE	21	CH	70	3400	2700	1.28
10	GE	21	MWH	70	3400	2600	1.32

**^a^** Measured by GPC in THF (40 °C) using PS standards and corrected by applying the correcting factor (0.56); **^b^**All monomer conversions >95%.

**Figure 5:**
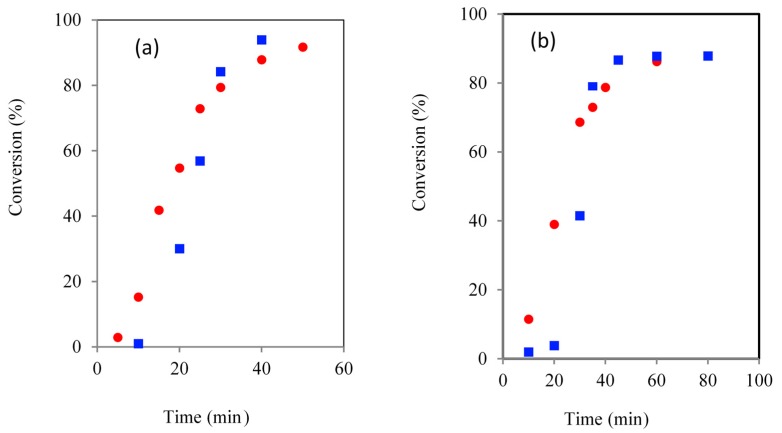
Comparison of TMPE initiated ROP kinetics using CH (■) and MWH (●) (150 °C, DP 90 (**a**) and 21 (**b**)).

The expected trends were observed from these reactions in that all the ethoxylated liquid physical form initiators demonstrated shorter overall reaction times compared to when solid form initiators were used. Whilst the comparison of the G and GE results showed that these were completed in comparative times (compare Table 1 and Table 3, and see Figure S4 ESI). This confirmed the link between the initiator’s physical form and overall reaction time. Additionally, the effect of changing the initiator’s molecular structure/physical form was observed to have the most significant effect upon those reactions that were targeting the lower DP’s. For example, the cycle time for DP 21 TMPE reaction was observed to be reduced by approximately 80% compared to the TMP equivalent, whilst the reduction was only around 60% for the same DP 90 ROP. However, in all the cases where ethoxylated initiators were used the CH reactions appear to have a reached overall conversation prior to the MWH reactions. Additional examinations of the kinetics of these polymerisations showed different trends than the case of the non-ethoxylated hydrocarbyl initiators whether they were of solid or liquid physical form, an example set of kinetic data for the TMPE reactions is shown in Figure 5.

Inspection of the kinetic plots showed that the induction periods of the TMPE and PTOLE the reactions had been significantly reduced compared to the TMP and PTOL equivalents Both MWH and CH TMPE experiments were noted to be significantly shorter than with TMP initiation, but the MWH reaction had been reduced to a greater extent than the CH cases (60 (DP 90)/90 (DP 21) min with MWH and 150 (DP 90)/180 (DP 21) with CH). MWH ROP induction periods with the ethoxylated TMPE and PTOLE initiators were always found to be typically 50%–60% shorter compared to the CH equivalents. For example, when MWH was used with TMPE, the DP 90 and DP 21 IPs were 5 and 10 min respectively, whilst those using CH were 15 and 20 min. Similar trends were demonstrated in PTOLE initiated reactions, where the IPs were reduced by a factor of approximately 4 for DP 90 and 5 for DP 21. In the case of the G/GE comparison it was observed that the CH induction time was very similar as both initiators are liquid and miscible, however in the MWH the GE initiation was slower than the G case. Thus this demonstrated that the steric environment and functional group reactivity (G contains two more reactive primary hydroxyls and one less reactive secondary, whilst GE has 3 primary) of these initiators in less important in governing the induction period than its physical/dielectric properties which will determine its propensity for producing the true catalytic species by reaction with the Sn(Oct)_2_.

The root cause behind the alignment in the MWH and CH overall cycle times (see Figure 5) was also highlighted by these kinetic plots. They were shown to describe a different trend at high conversion when compared to those of their non-ethoxylated equivalents. When utilising hydrocarbyl initiators, the propagation rates as defined by the slope of the linear relationship when plotting conversion against time, was always observed to be similar to that of the CH equivalents. Meanwhile, with all the ethoxylated alternatives, whilst the induction and initial, low conversion propagation stages of the reaction profiles were still faster with MWH. At higher conversion, the MWH propagation rate was observed to reduce and move away from the ideal linear relationship earlier than the CH cases. These observations suggested that the MWH polymerisation conducted with ethoxylated precursors was also differentiated at higher conversion, not just during initiation.

The key molecular difference between the initiators under study was the presence/absence of ethoxylate arms. However, it was thought unlikely that steric effects related to these small extended arms on the initiator would affect either the polarisation (electromagnetic) or polymerisation (chemical) mechanisms adopted at the high conversions. This is because the reaction centres will be at the opposite end of the lengthy PCL chain by the time this difference was observed. Therefore, this effect was attributed to changes in the extended macro-structure within the reaction mixture resulting from the material properties of the amphiphilic core corona star polymers themselves. The initial ethoxylate arms are hydrophilic in character and the PCL arms growing via ROP are regarded as being hydrophobic. Hence, by growing the PCL arm extensions, a central hydrophilic region is surrounded by a larger corona of hydrophobic polymer. It was proposed that this may lead to either the generation of extended order within the reaction mixture. The most likely of which was the development of “pseudo-micellar” behaviour by the amphiphilic star polymer, with the PCL sections forming loops about the ethoxylate central core. As a result, the hydrophilic hydroxyl dormant chain end prefers to locate itself within internal ethoxylate core of the “micelle” and, as such, they are separated from the free monomer and Sn catalyst and will find increased difficulty in achieving propagation. This conclusion was supported by inspection of the temperature profiles of the ethoxylate polymerisations. These showed that the temperature of the MWH reactions dropped at higher conversion whilst the CH did not. This supported by the proposal that the MWH systems are affected more than CH in this region by the evolution of the core corona star structure. It indicated that the differences in reaction kinetics achieved by selective MWH heating of the Sn species is more difficult to achieve with these core-corona structures. This was linked to the increased molecular interaction/structure resulting in both partitioning of the reagents and/or a reduced ability/efficiency of the dipoles to interact with the incident microwave energy [40,41,42,43]. The observation that the ROP synthesis using GE as initiator exhibited the same trend both supported the conclusion that this effect was not related to the change from in initiator physical form/dielectric properties and therefore that the extended structure hypothesis was valid.

Thus, whilst the use of ethoxylated, amphiphilic initiators had been successfully shown to produce well controlled, amphiphilic core corona star polymers containing a hydophilic core and a hydrophobic outer shell, no overall cycle time advantage was gained from applying MWH alone. Rather, the best strategy to improve the CT of such amphiphilic stars was proposed to be where the ROP is initiated using MWH and completed using CH. Such a reaction was conducted and noted to reduce the overall CT from 70 to 50 min. Additionally, the fact that these kinetic pathway differences were found to be consistent with all non-ethoxylated and ethoxylated initiators despite the physical form, steric environment or functional group reactivity, supported the conclusion that this change in MWH propagation rate was primarily related to the macro-structuring in the reaction medium. This takes effect as the molecular size of the hydrophobic corona increases producing a PCL segment long enough to create a loop back to the core.

**Figure 6:**
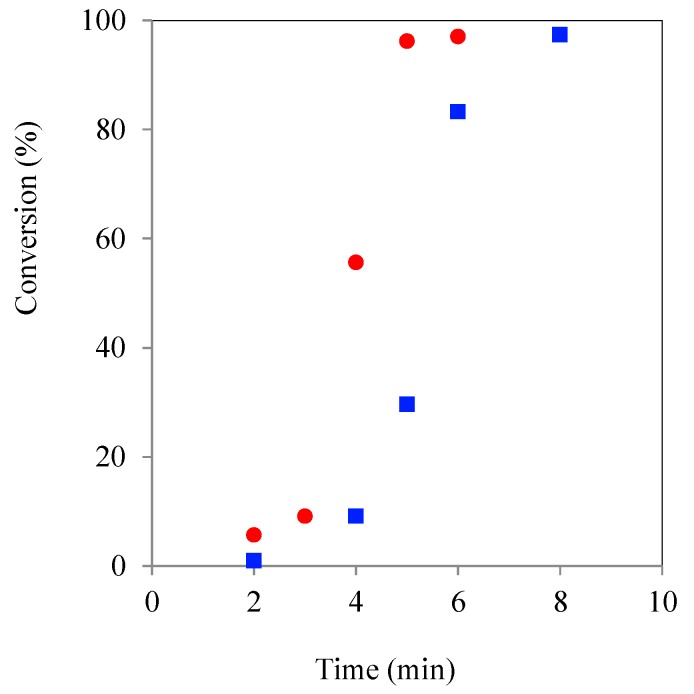
Plot of TMPE ROP initiated kinetic experiment using CH (■) & MWH (●) (150 °C, DP 90) with CL/Sn(Oct)_2_ = 1:2.87 x 10^−3^.

Finally, experiments were conducted with an increased concentration of tin precatalyst to define if this alteration would overcome the catalyst partitioning problems proposed to exist in the “micelle segregated” systems in the amphiphilic core corona stars systems. These experiments attempted to further reduce the induction time and increase the high concentration propagation rate of core corona star MWH synthesis by overcoming the partitioning by increasing the level of selective heating achieved in the MWH systems. Consequently, the catalyst loading was increased by an order of magnitude (CL/Sn(Oct)_2_ = 1:2.87 × 10^−3^) and kinetic studies were performed targeting a DP = 90, see Figure 6.

Comparison of the plots in Figure 6 with Figure 5, showed that the increase in tin concentration had produced overall reaction time reductions (MWH = 50 to 5 min and CH = 40 to 8 min), as would be expected due to the higher tin concentration from literature reports [34]. Furthermore, the MWH profile was found to no longer drop behind the CH profile at higher conversions and so gave an aligned overall cycle time. Therefore, it was concluded that the greater quantity of selectively heated tin moieties has overcome any reaction medium/molecular structure induced retardation of the polymerisation rate. This supports the hypothesis that the retardation effects reported at the lower catalyst concentrations are related to partitioning/diffusion issues that have been rectified by increasing the availability of the selectively heated species. Additionally, because single and narrow distribution GPC spectra were again observed for the product polymers obtained from these higher catalyst formulations, these experiments also indicated that both hydrophobic and core corona stars could be synthesised in a reaction time between 2 and 20 min (Figure S5, ESI). A more detailed investigation of the structures produced by the two heating methods and different reaction times and initiator types is currently under way and will be the subject of a subsequent literature report.

## 3. Experimental Section

### 3.1. Materials

All chemicals were used as received without further purification. ε-caprolactone (99%) was purchased from Acros (Geel, Belgium). Karl-Fisher titration determined its water content to be 67 ppm. Tin 2-ethylhexanoate (96%), pentaerythritol (98%), glycerol (99.5%), glycerol ethoxylate (~1000 Da), pentaeryhtitol ethoxylate (~270 Da) and trimethylolpropane ethoxylate (~450 Da) were purchased from Sigma-Aldrich (Dorset, UK) and tmethylolpropane (98%) was purchased from Alfa Aesar (Lancashire, UK).

### 3.2. Characterization—Nuclear Magnetic Resonance (NMR) Analysis

^1^H-NMR spectra (300 MHz) were recorded in CDCl_3_ using a DPX-300 spectrometer (Bruker, Ettlingen, Germany) for kinetic/non-precipitated samples and a 400 MHz Bruker DPX-400 spectrometer for purified polymers. Chemical shifts are reported relative to SiMe_4_ and were determined by reference to the residual ^1^H solvent peak. Number-average Mwt (Mn) was determined by end-group analysis by comparison of the measured integrals of the methylene proton resonance adjacent to the carbonyl group (δ = 4.1 ppm) and that of the methylene proton of the initiator fragment located at the centre of the star (in linear polymers this benzyl ester end-group resonance is ~δ = 5.1 ppm). Monomer conversion was determined by comparing the integral of the proton resonance of the methylene moiety adjacent to oxygen of the carbonyl group for both the monomer (-CH_2_OCO-, δ = 4.24 ppm) and polymer (-CH_2_OCO-, δ = 4.07 ppm).

### 3.3. Gel Permeation Chromatograph (GPC) Analysis

GPC was performed on a GPC-120 instrument (Polymer Labs, Stretton, UK) equipped with a PLgel 5 mm guard column and two 30 cm PolarGel-M columns in series coupled with a refractive index detector. The samples used HPLC grade THF as the mobile phase at a flow of 1.0 cm^3^·min^−1^, and were performed at 40 °C and typically took 24.5 min. The GPC calibrated using poly(styrene) (PS) standards ranging from 580 to 377,400 g·mol^−1^. All GPC equipment and standards were supplied by Polymer Labs. GPC data were analysed using the Cirrus GPC Offline software package (3.0, Marlow, UK). Typically, a polymer solution of 7 mg/mL PCL in HPLC grade THF was prepared, and filtered through a 0.2 µm sieve into a GPC vial in preparation for analysis. For the direct assessment of the product Mwt, samples were precipitated in MeOH prior to GPC analysis. For kinetic experiments, samples of the crude polymerisation mixture were diluted to the appropriate concentration, filtered and injected without further purification. A correction factor of 0.56 was applied to the final Mwt predictions to allow for the differences in the hydrodynamic volumes of the sample and standard polymers [37,44,45].

### 3.4. Dielectric Property Analysis

The response of a material to microwaves can be quantified by the dielectric properties of that material. In this study these properties were measured in order to develop understanding of the interaction between microwave energy and the materials within each component in the reaction mixture. The dielectric properties are related to the complex permittivity, ε*, which is a combination of the real and imaginary parts. The real part is known as the dielectric constant, ε’, and is related to the ability of a material to be polarised and store electromagnetic energy through polarisation. The imaginary part is termed the dielectric loss factor, ε″, which expresses the ability of that material to convert the stored electromagnetic energy to heat. The loss tangent (tanδ) is defined as the ratio of dielectric loss and dielectric constant, which can be used to quantify the extent to which a material heats in an applied microwave field [46,47,48]. The dielectric properties of the polymerisation components were determined using both cavity perturbation and open-ended coaxial probe techniques as described in previous publications [40,41]. Both techniques were used to assess the dependence of the component’s dielectric properties on temperature and frequency. The cavity perturbation technique is only suited for low loss materials [49]. The open-ended coaxial probe technique is a versatile method that can produce measurements across a swept frequency range (100 MHz to 20 GHz) and is best suited for medium to higher loss materials [43,48,49,50].

### 3.5. Typical Synthetic Polymerisation Procedures

All polymerisations were conducted in bulk. For a target DP of 90, CL (90 equivalents) and the appropriate initiator (1 equivalents) were introduced into a flask. Sn(Oct)_2_ catalyst (either 0.05 g or 0.51 g of a 4.98 × 10^−1^ g/mL stock solution for low and high tin concentrations respectively) was then added via syringe to form a CL/Sn(Oct)_2_ ratio of either 1:2.80 × 10^−4^ or 1:2.87 × 10^−3^. The flask was then equipped with a magnetic stirrer bar, sealed and heated for the set reaction time at 150 °C by either a pre-heated oil bath or a microwave reactor (CEM Discover SP microwave reactor, Matthews, NC, USA) maximum output power of 300 W, operating at a frequency of 2450 MHz). Reaction temperatures were assessed and controlled by a direct measurement of the reaction bulk via a fibre optic probe inserted into the reaction medium. Other DP’s were obtained by following the same method and adjusting the monomer to initiator ratio accordingly. Synthetic kinetic experiments involved taking sequential samples for analysis at set time periods throughout the reaction to follow polymerisation progress with time in terms of conversion, Mwt and PDI.

## 4. Conclusions

This paper has demonstrated that the synthesis of both high quality (*i.e.*, close to target Mwt and low PDI) 3- and 4-arm hydrophobic homo-polymer and amphiphilic core corona star polymers can be achieved via controlled ROP using Sn(Oct)_2_ and multi-ol initiators. It has demonstrated that the key factor that results in the elongation of reaction times in star polymer synthesis, when compared to equivalent linear polymerisations, is a significant lengthening of the reaction induction time. Furthermore, the primary factor that determines the length of the induction time are the physical/dielectric properties of the initiator used because this determines the availability of the initiating species to undergo the “*in-situ*” formation of the true catalytic species with the Sn(Oct)_2_ precursor. This was observed to excerpt far greater influence over the induction times exhibited than the reactivity or steric environment of the hydroxyl groups on the initiator. Consequently, by ensuing the initiators used are of liquid physical form and/or highly miscible with the monomer of choice, the cycle time required to produce these star species can be reduced from several hours to several minutes.

When comparing the use of microwave and conventional heating methods. All the ROPs to form hydrophobic stars were observed to require less reaction time when conducted with MWH. This was observed to be primarily due to a significant further reduction in the induction period attributed to the selective heating the tin containing reaction components. This resulted in the reaction temperature being reached more rapidly and the “*in-situ”* catalyst formation occurring at a higher rate than the CH equivalents, as has been reported in similar linear polymerisations because the tin species are hotter locally than is predicted by the bulk temperature measurement.

In the case of the amphiphilic core corona stars, at the lower tin concentrations, the MWH reactions exhibited a shorter induction time but an overall cycle time that was similar to or longer than the CH methods. This was determined to be due to the MWH reactions exhibiting a slower propagation rate that the CH ROPs with these initiators, particularly at higher conversions. The slower/reduction of the propagation rate at high conversion was attributed to macro structuring in the reaction medium as the corona builds in size. This macro-structuring leads to a portion of the dormant, hydrophilic hydroxyl-terminated chain being located within the hydrophilic core once the PCL arm is long enough to create a loop back to the core. Thus it becomes partitioned away from the available Sn species and the monomer. This macro-structuring therefore reduced the positive attributes of MWH selective heating of the tin species by isolating the hydroxyl chain end reducing its availability for reaction. By comparison the more “random” bulk convection heating mechanisms of CH will introduce convection currents that help disrupt this macro ordering, and so it does not exhibit a propagation rate reduction at high conversion. Therefore, it was concluded that (a) macrostructure within the reaction medium can influence the efficiency of which MWH selective heating can influence chemical transformation; and (b) the best heating strategy for fast synthesis of core corona stars at low Sn concentrations was through initiation by MWH and propagation by CH. This conclusion was supported by the application of higher tin concentrations which overcame the partitioning problems by increasing the concentration of tin species and so the influence of MWH selective heating within the amphiphilic ROPs. This resulted in reduced cycle times for MWH ROPs because not reduction in propagation rate was observed at higher conversions. This confirmed conclusions made in earlier publications about the need to achieve critical concentrations of selective heating species within systems to ensure that a differentiated microwave effect is achieved [34].

## Supplementary Materials

Supplementary materials can be accessed at: http://www.mdpi.com/1420-3049/20/11/19681/s1.
