# Supplementary material for: Molecular Differentiated Initiator Reactivity in the Synthesis of Poly(caprolactone)-Based Hydrophobic Homopolymer and Amphiphilic Core Corona Star Polymers
Source: Molecules. 2015 Nov 9;20(11):20131–45. doi: 10.3390/molecules201119681 (PMC6332146; doi:10.3390/molecules201119681)
Supplement: Supplementary file 1 [file molecules-20-19681-s001.pdf]

Supplementary Material: Molecular Differentiated Initiator Reactivity in the Synthesis of Poly(caprolactone)-Based Hydrophobic Homopolymer and Amphiphilic Core Corona Star Polymers

Eileen Deng, Nam T. Nguyen, Frédéric Hild, Ian E. Hamilton, Georgios Dimitrakis, Samuel W. Kingman, Phei-Li Lau and Derek J. Irvine

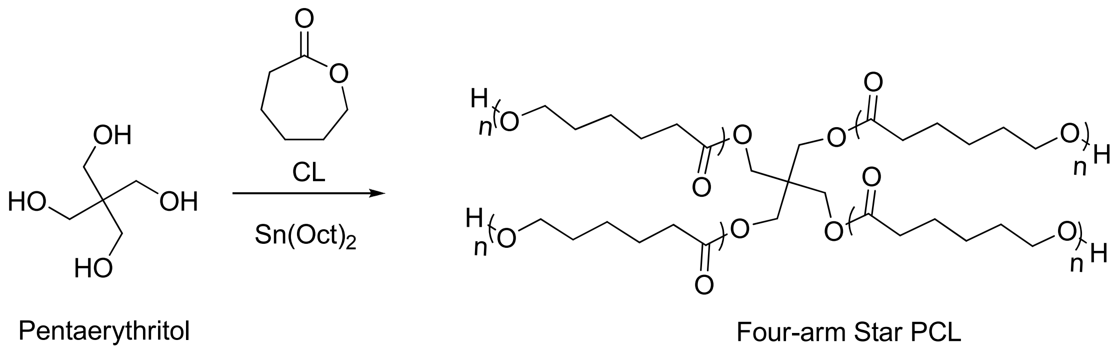

Scheme S1. Synthesis of four-arm star PCL using CH and MWH with pentaerythritol (PTOL) and $Sn(Oct)_2$ as initiator/catalyst system.

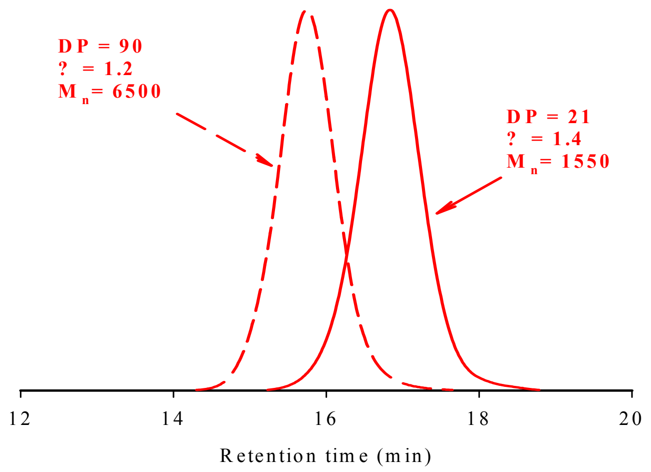

Figure S1. GPC traces of 3-arm star PCL (DP = 90 and DP = 21) initiated by TMP.

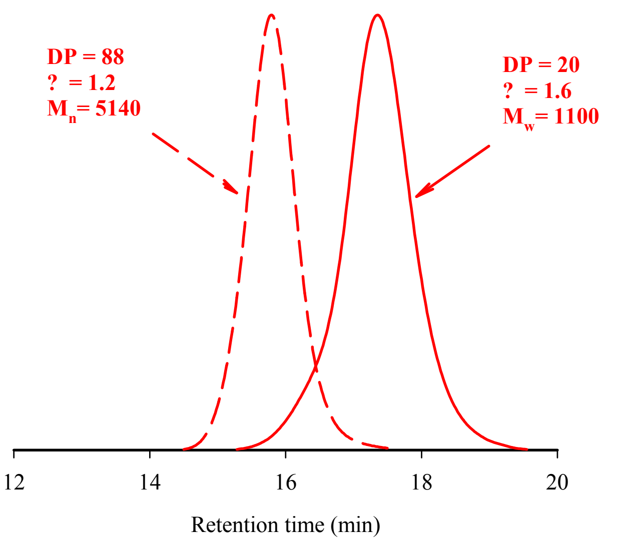

Figure S2. GPC traces of 4-arm star PCL (DP = 88 and DP = 20) initiated by pentaerythritol (PTOL).

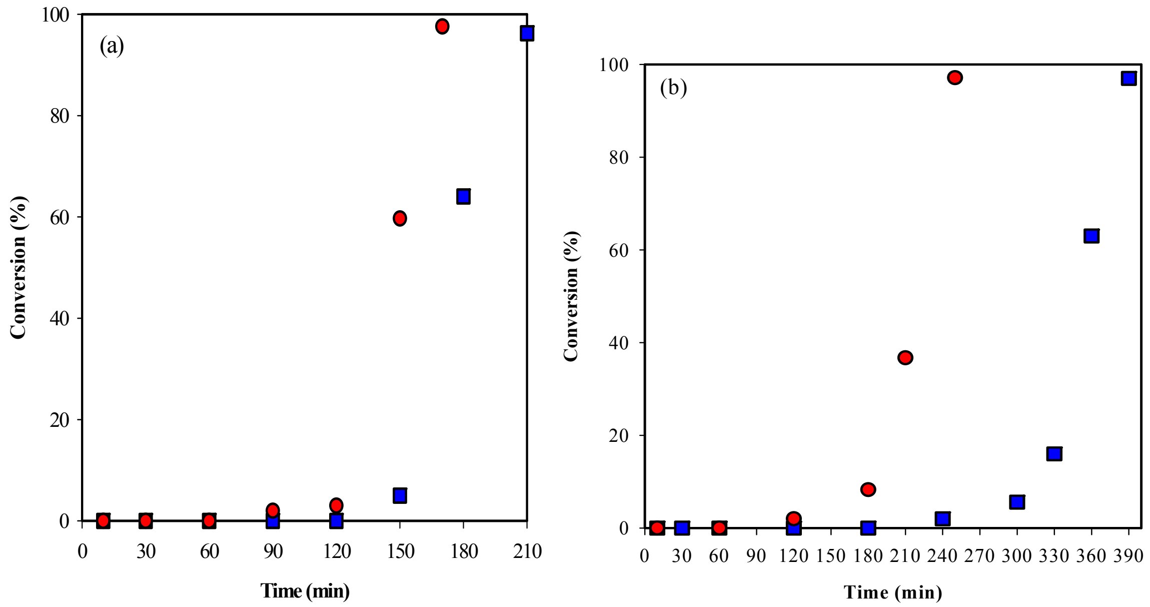

Figure S3. Comparison of the kinetics of the ROP initiated with PTOL, conducted in a round bottom flask using CH (■) and MWH (●) (150 °C, DP (**a**) 88 and (**b**) 20).

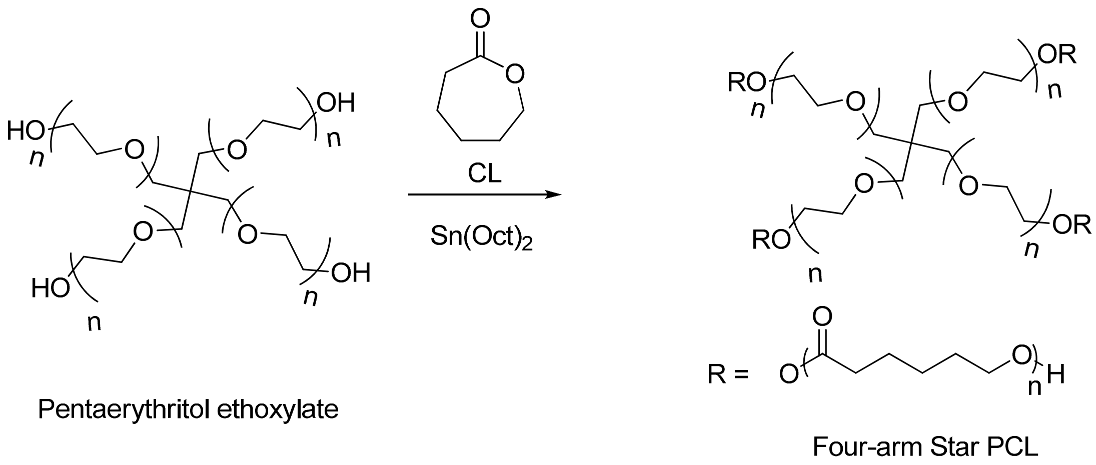

Scheme S2. Synthesis of four-arm star PCL using CH and MWH with PTOLE and $Sn(Oct)_2$ as initiator/catalyst system.

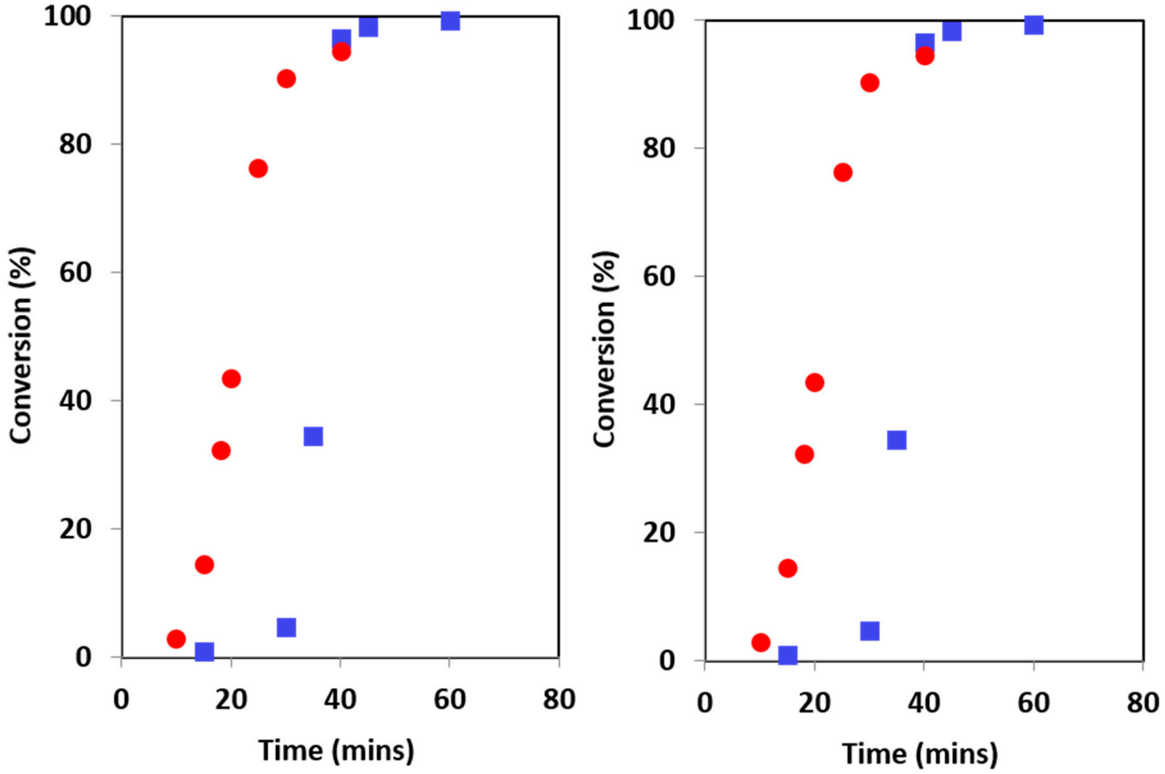

Figure S4. Comparison of G (left) and GE (right) initiated ROP the kinetics using CH (■) and MWH (●) (150 °C, DP90).

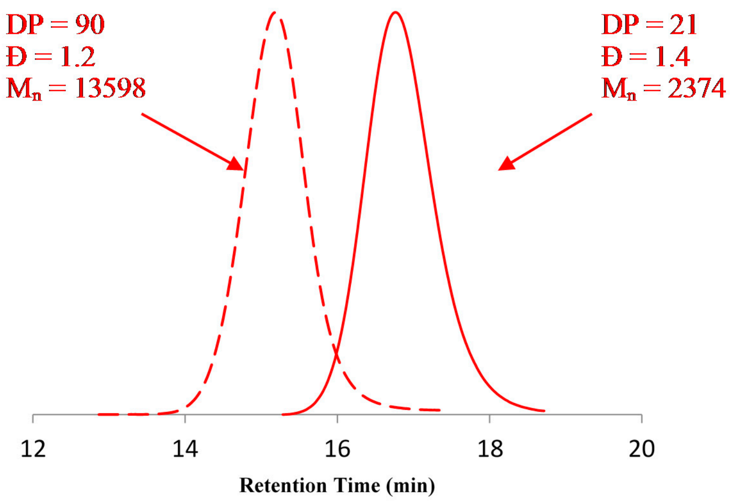

Figure S5. GPC traces of 3-arm star PCL (DP = 90 and DP = 21) initiated by TMPE.
